# Serendipity, opportunity and impact

**DOI:** 10.4102/sajid.v37i2.401

**Published:** 2022-02-14

**Authors:** Marc Mendelson

**Affiliations:** 1 Division of Infectious Diseases & HIV Medicine, Department of Medicine, Faculty of Health Science, University of Cape Town, Cape Town, South Africa

A career is more about opportunity and serendipity than ‘wisdom’. I do not believe that there is one perfect career path but a series of options that will take you on different adventures. I think embracing that may be more productive than worrying whether you have made the right choice.

I failed and re-took A levels and entered the only medical school to give me an offer based predominantly on my ability to play rugby. I was interested in infectious diseases (ID) as my studies coincided with the first cases of the emerging HIV epidemic in London.

HIV had piqued my interest in cytomegalovirus, and I followed the footsteps of a mentor to Cambridge for a molecular biology-based PhD. This is not for everyone. Being a clinician and a laboratory scientist are very different activities, and precious few end up carrying out both efficiently. I certainly did not. In the South African context, I would suggest public health or clinically based research degree if you are interested in academic ID.

During training in ID, I became interested in tuberculosis (TB), having had a series of patients with intractable cerebral tuberculomas requiring the rarely used thalidomide. I looked around for people working in TB and was told about a specialist in Cape Town called Gary Maartens. We exchanged emails and ideas; however, funding was a problem. Opportunity knocked as Gary was collaborating with Gilla Kaplan at the Rockefeller University in New York, who was interested in thalidomide analogues. In 2001, I spent a year with Gilla working on TB but not thalidomide, as I was in the lab of Ralph Steinman, who later received the Nobel Prize for his discovery of the dendritic cell (DC). I became sidetracked. After a year, I moved to Cape Town to work with Gary and Gilla on clinical studies with TB and blood DCs.

Two years became five, and ID became a recognised sub-speciality of Medicine in South Africa. With the support of Bongani Mayosi, the University of Cape Town (UCT) proposed a training unit to be set up at Groote Schuur Hospital (GSH). In 2007, we started building the ID unit at GSH. It has been a singular honour, a wonderful experience and a rare opportunity to build a speciality at UCT. I have made many, many mistakes, but hopefully got some things right and have contributed to expanding the pool of ID sub-specialists in South Africa over the years. I owe so much to Gary, Bongani and more recently to my once registrar, Ntobeko Ntusi, as well as all whom I have worked with and supported me.

In terms of specific advice, I do not have the blueprint! I would opt for a portfolio career. Every job has its ‘bread and butter’ tasks, which are generally dull and drive you mad. Different themes within your day – clinical, research, teaching, policy – help and stimulate you. Be ready to develop new interests or react to the changing landscape. For me, this was antibiotic resistance and, more recently, coronavirus disease 2019 (COVID-19).

It is natural to say yes to every opportunity; however, after the first few years of your consultancy, pause and reflect. If you spread yourself too thin, you will run into problems. If you say no too much, people will lose interest. So, work out what brings you joy and stimulation and focus your yeses on that. Learning how to say no kindly takes practice.

Do not try to be too much like your mentor. Be yourself and develop your own interests in ID. If you constantly worry that you do not live up to your mentors, you are in for a rough ride. It is okay to be different!

Find an opportunity to experience ID outside of your local context. There are various fellowships such as the European Society of Clinical Microbiology and Infectious Diseases (ESCMID) that can take you abroad. There is no fixed period, and it will depend on your family responsibilities, finances, and so on; however, bringing new skills back to South Africa can be very valuable to you and the country.

Advocate for something that you are passionate about. Sometimes, it is good to stick your head above the parapet. As an ID specialist, you can stimulate change. If you do not enjoy leading from the front, join a passionate cause that others lead. Find a home in professional societies that share your interest. International discussions on ID are often dominated by practitioners and scientists from high-income countries. As a clinician in a middle-income country with significant resource limitations, your voice is extremely valuable. Advocate through publications. Impact factors are great but think of the reach your opinions could get through other routes. During the COVID-19 pandemic, online publications, such as the Daily Maverick, GroundUp, The Conversation and others, have extensive reach and helped the public understand our speciality.

Whatever your path, I wish you all success and that it will bring you happiness. Remember, there is no perfect way to do things.

